# Staphylococcal Panton–Valentine Leucocidin and Gamma Haemolysin Target and Lyse Mature Bone Marrow Leucocytes

**DOI:** 10.3390/toxins12110725

**Published:** 2020-11-20

**Authors:** Elisabeth Hodille, Adriana Plesa, Eve Bourrelly, Lucie Belmont, Cédric Badiou, Gerard Lina, Oana Dumitrescu

**Affiliations:** 1 Department of Bacteriology, Hospices Civils de Lyon, Hôpital de la Croix-Rousse, Centre de Biologie Nord, 69004 Lyon, France; gerard.lina@univ-lyon1.fr (G.L.); oana.dumitrescu@chu-lyon.fr (O.D.); 2 Centre International de Recherche en Infectiologie (CIRI), INSERM U1111, CNRS UMR5308, ENS Lyon, Université Lyon 1, 69364 Lyon, France; cedric.badiou@univ-lyon1.fr; 3 Department of Hematology, Hospices Civils de Lyon, Centre Hospitalier Lyon Sud, Centre de Biologie Sud, 69002 Lyon, France; adriana.plesa@chu-lyon.fr (A.P.); evebourrelly@hotmail.fr (E.B.); lucie.belmont@gmail.com (L.B.); 4 National Reference Center for Staphylococci, Hospices Civils de Lyon, Hôpital de la Croix-Rousse, Centre de Biologie Nord, 69004 Lyon, France

**Keywords:** *Staphylococcus aureus*, Panton–Valentine leucocidin, gamma haemolysin, bone marrow leucocytes, necrotising pneumonia

## Abstract

*Staphylococcus aureus* is a major human pathogen, inducing several infections ranging from the benign to the life-threatening, such as necrotising pneumonia. *S. aureus* is capable of producing a great variety of virulence factors, such as bicomponent pore-forming leucocidin, which take part in the physiopathology of staphylococcal infection. In necrotising pneumonia, Panton–Valentine leucocidin (PVL) induces not only lung injury and necrosis, but also leukopenia, regarded as a major factor of a poor prognosis. The aim of the present study was to evaluate the effect of bicomponent pore-forming leucocidin, PVL and gamma haemolysin on bone marrow leucocytes, to better understand the origin of leukopenia. Using multi-parameter cytometry, the expression of leucocidin receptors (C5aR, CXCR1, CXCR2, and CCR2) was assessed and toxin-induced lysis was measured for each bone marrow leucocyte population. We observed that PVL resulted in myeloid-derived cells lysis according to their maturation and their C5aR expression; it also induced monocytes lysis according to host susceptibility. Haemolysin gamma A, B, and C (HlgABC) displayed cytotoxicity to monocytes and natural killer cells, hypothetically through CXCR2 and CXCR1 receptors, respectively. Taken together, the data suggest that PVL and HlgABC can lyse bone marrow leucocytes. Nevertheless, the origin of leukopenia in severe staphylococcal infection is predominantly peripheral, since immature cells stay insensitive to leucocidins.

## 1. Introduction

*Staphylococcus aureus* is a major human pathogen, resulting in a plethora of infections that range from superficial skin infections to severe invasive diseases [1]. *S. aureus* pathogenesis involves the production of toxins such as the staphylococcal bicomponent pore-forming toxins Panton–Valentine leucocidin (PVL) and gamma haemolysin (Hlg). Such toxins are composed of an S sub-unit (LukS-PVL, HlgA or HlgC) and a F sub-unit (LukF-PVL or HlgB) [2]. The *hlg* gene is present in the core genome of *S. aureus* species and encodes two different bicomponent pore-forming toxins (HlgAB and HlgCB), whereas the *pvl* gene is carried by phages and is inserted only in a subset of strains [2].

Recently, it has been reported that the staphylococcal bicomponent pore-forming toxin target specifically subsets of human cells through the binding of the S sub-unit to its human receptor; PVL and HlgCB both induce human phagocyte cell death via interaction with the complement receptors C5aR and C5L2 [3,4], and HlgAB does so via CXCR1, CXCR2, and CCR2 [4]. In addition, Tromp et al. demonstrated that the targeting of human cells was also mediated by the binding of the F sub-unit, in particular lukF-PVL, which specifically targeted human CD45 [5].

With regard to the pathogenesis of staphylococcal infections, there is strong evidence supporting that PVL-producing *S. aureus* is associated with staphylococcal life-threatening necrotising pneumonia [6]. Interestingly, the onset of leukopenia during necrotising pneumonia has been identified as a major risk factor for mortality [7]. Based on PVL and Hlg activities, the current model of leukopenia during necrotising pneumonia in rabbits involves the attraction of polymorphonuclear neutrophil cells (PMNs) to the lung and their subsequent destruction by staphylococcal leucocidins [8]. Nevertheless, no study has analysed their potential effect on human bone marrow leucocytes, while, in the past, Szmigielski et al. did show that PVL injection in rabbits resulted in an initial decrease in granulocytic leucocyte number in the blood and in the bone marrow, before an increase [9].

The aim of the present study was to examine the impact of PVL and HlgABC on bone marrow leucocytes, and the potential relationship with the expression of staphylococcal leucocidin receptors. We report that PVL triggers the lysis of bone marrow PMN and granulocytic immature 2 (Gr2) cells, but not granulocytic immature 1 (Gr1) cells. PVL action on the myeloid line was associated with the differential C5aR expression between subpopulations of cells. Moreover, we observed that HlgABC challenge resulted in the lysis of CD11b+ monocytes and natural killer cells (NK). Taken together, the results strengthen the hypothesis regarding the foremost peripheral origin of the leukopenia observed during necrotising pneumonia.

## 2. Results

### 2.1. CXCR1, CXCR2, CCR2, and C5aR Expression on Different Bone Marrow Leucocyte Subpopulation

**Figure 1:**
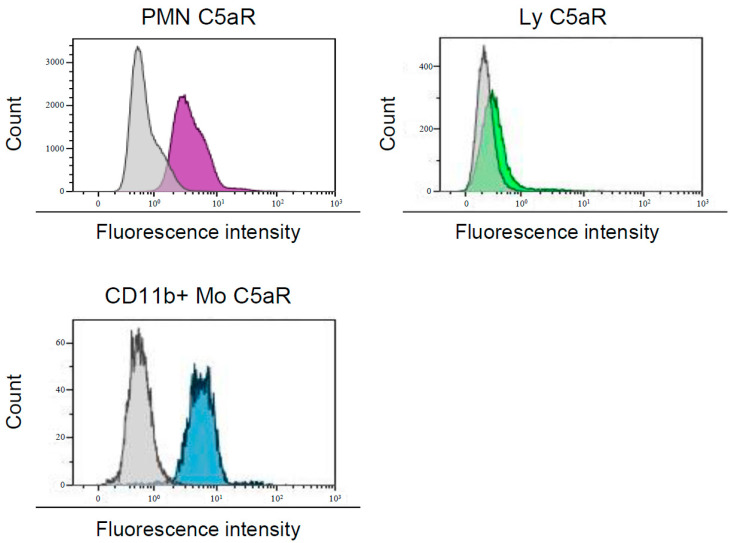
Analysis of C5aR expression on bone marrow leucocytes subpopulation. Expression was assessed using monoclonal antibodies coupled to fluorescein isothiocyanate (FITC) compared to isotypic control. The ratio median fluorescence intensity (RMFI) is the ratio between the median of the intensity of the fluorescence of specific monoclonal antibodies (purple for polymorphonuclear neutrophil cells (PMNs), green for Ly and blue for CD11b+ Mo) and the median of the intensity of the fluorescence of the isotypic control (grey). PMN, polymorphonuclear neutrophil cells; Ly, lymphocytes; CD11b+ mo, CD11b+ monocytes.

Receptor expression was assessed by the ratio of median fluorescence intensity (RMFI), being the ratio between the median of the intensity of the fluorescence of specific monoclonal antibodies and the median of the intensity of the fluorescence of the isotypic control (Figure 1). RMFI ≥ 2 indicated that the population expressed the receptor. For each bone marrow leucocyte population, the mean of RMFI observed in the 11 bone marrows was calculated for the receptors CXCR1, CXCR2, CCR2, and C5aR. Expression of CD45 was not assessed by RMFI, since all studied populations of bone marrow leucocytes were gated on CD45 expression.

**Figure 2:**
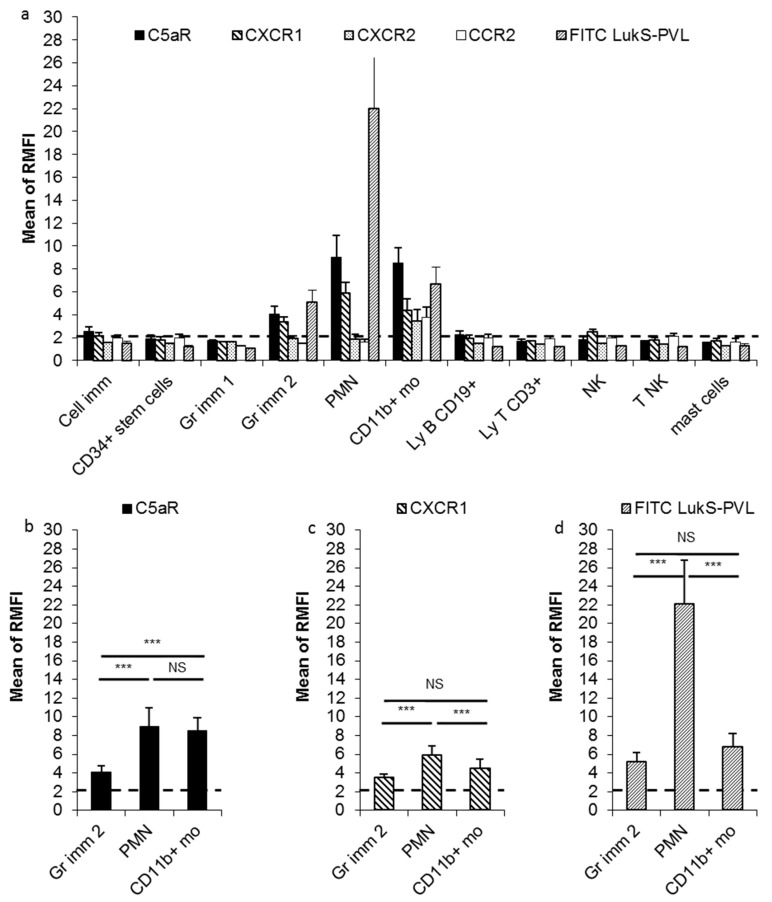
Expression of receptors on bone marrow cell populations. (**a**) C5aR, CXCR1, CXCR2, CCR2 expression, and fluorescein isothiocyanate (FITC) LukS-Panton–Valentine leucocidin (PVL) binding on bone marrow leucocyte surface. Expression levels were assessed by the ratio of the median fluorescence intensity of a specific monoclonal antibody/median fluorescence intensity of the control (RMFI). RMFI ≥ 2 indicated that the population expressed the receptor. (**b**) C5aR expression on the granulocytic myeloid-derived cells and CD11b+ monocytes. (**c**) CXCR1 expression on the granulocytic myeloid-derived cells and CD11b+ monocytes. (**d**) FITC LukS-PVL binding on the granulocytic myeloid-derived cells and CD11b+ monocytes. Cell imm, immature cells; Gr imm 1, granulocytic immature 1 cells; Gr imm 2, granulocytic immature 2 cells; PMN, polymorphonuclear neutrophil cells; CD11b+ mo, CD11b+ monocytes; LyB CD19+, CD19 B lymphocytes; Ly T CD3+, T lymphocytes; NK, natural killer; T NK, T natural killer. The mean value of 11 bone marrows is shown; error bars correspond to standard deviation. Comparisons made using Student’s *t*-test with Bonferroni adjustment, *** *p* < 0.001; NS, non-significant.

CXCR1, CXCR2, CCR2, and C5aR expression (considered as positive when ≥2x the level of istotype control) varied among bone marrow leucocyte subpopulations; CD34+ stem cells, Gr1 cells, T lymphocytes, and mast cells did not express any receptors. Immature cells displayed a low level of C5aR expression (mean ± standard deviation, SD: RMFI 2.53 ± 0.45) and CXCR1 (RMFI 2.19 ± 0.28). Gr2 cells and PMN expressed of C5aR (RMFI 4.09 ± 0.67 and 9.01 ± 1.93, respectively) and CXCR1 (RMFI 3.41 ± 0.40 and 5.88 ± 0.98, respectively). CD11b+ monocytes expressed all tested receptors: RMFI 8.54 ± 1.35 for C5aR, 4.41 ± 1.00 for CXCR1, 3.47 ± 1.00 for CXCR2, and 3.76 ± 0.89 for CCR2. CD19+ B lymphocytes expressed a low level of C5aR (RMFI 2.23 ± 0.32). NK and T NK expressed a low level of CXCR1 (RMFI 2.49 ± 0.23) and CCR2 (RMFI 2.10 ± 0.28; Figure 2a) respectively.

In the granulocytic myeloid-derived cells, PMN expressed significantly more C5aR receptors than Gr2 cells and more of CXCR1 receptor than Gr2 cells and CD11b+ monocytes (*t*-test, *p* < 0.001; Figure 2b,c); C5aR expression on CD11b+ monocytes was significantly higher than on Gr2 cells (*t*-test, *p* < 0.001; Figure 2b).

### 2.2. Fluorescein Isothiocyanate (FITC) LukS-PVL Binding to Different Bone Marrow Leucocyte Populations

Fluorescein isothiocyanate (FITC) LukS-PVL was bound to Gr2 cells (mean ± SD: RMFI 5.09 ± 1.06), PMN (22.03 ± 4.71), and CD11b+ monocytes (RMFI 6.71 ± 1.45; Figure 2a,d). FITC LukS-PVL did not bind to CD19+ B lymphocytes (RMFI 1.20 ± 0.05) or immature cells (RMFI 1.48 ± 0.23), which expressed a low level of C5aR. FITC LukS-PVL binding to PMN was significantly higher than to Gr2 cells (*t*-test, *p* < 0.001; Figure 2d), and for both granulocytic myeloid-derived cells (Gr2 and PMN) FITC LukS-PVL binding was significantly correlated to C5aR expression (Pearson test, r = 0.92; 95% confidence interval (_95%_CI): (0.81; 0.97), *p* < 0.001; Figure S1a). There was no significant correlation between FITC LukS-PVL binding and C5aR expression on CD11b+ monocytes (Pearson test, *p* = 0.17; Figure S1a). Although PMN and CD11b+ monocytes had a similar level of C5aR expression (Figure 2b), FITC LukS-PVL binding to PMN was significantly higher than to CD11b+ monocytes (*t*-test, *p* < 0.001; Figure 2d). Likewise, despite the significantly higher C5aR expression on CD11b+ monocytes compared to Gr2 cells (Figure 2b), there was no significant difference in FITC LukS-PVL binding (Figure 2d).

### 2.3. Effect of PVL and HlgABC on Bone Marrow Leucocyte Sub-Population

**Figure 3:**
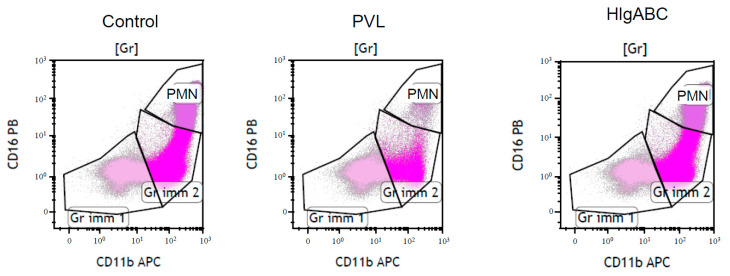
Gating strategy to assess toxins effect. The proportion of intact cells after toxins challenge was the ratio between the viable cells in the gate without toxins (Control) and the viable cells in the gate after challenge with 0.5 µg/mL of Panton–Valentine leucocidin (PVL), or with 0.1 µg/mL of haemolysin gamma A, B, and C (HlgABC). Gr, granulocytic myeloid-derived cells (Gr); Gr imm 1, granulocytic immature 1 cells; Gr imm2, granulocytic immature 2 cells; PMN, polymorphonuclear neutrophil cells; CD11b APC, anti-CD11b allophycocyanin; CD16 PB, anti-CD16 Pacific Blue.

To determine the cytotoxic effect of toxins on bone marrow leucocyte populations, we calculated the proportion of intact cells of each leucocyte sub-population after challenge with toxins, PVL (0.5 µg/mL lukS-PVL + 0.5 µg/mL lukF-PVL) or HlgABC (0.1 µg/mL HlgA + 0.1 µg/mL HlgB + 0.1 µg/mL HlgC), and expressed the results as a ratio compared to those without challenge (Figure 3).

#### 2.3.1. Effect of PVL

**Figure 4:**
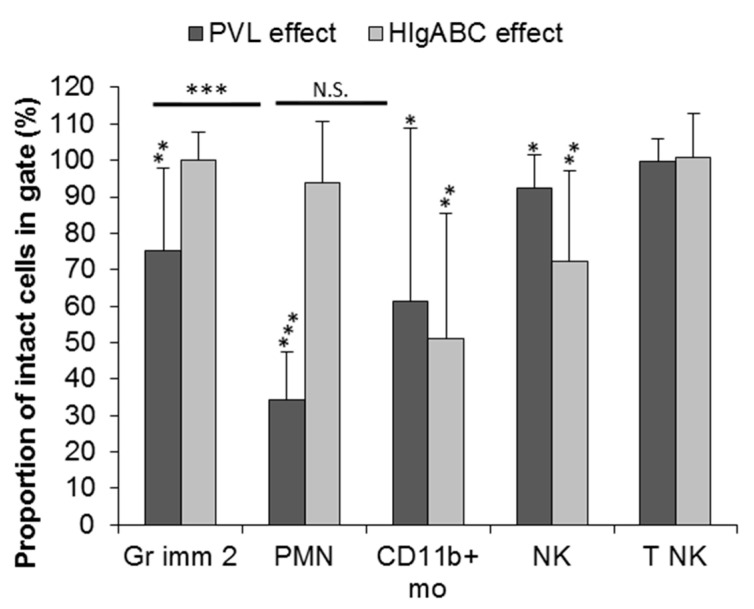
Effect of staphylococcal leucocidins PVL and HlgABC on bone marrow leucocytes, represented by the proportion of intact cells after incubation with toxins on the corresponding gate. Mean ± SD of 11 bone marrows for PVL and 10 bone marrows for HlgABC. For each sub-population, the effect of staphylococcal leucocidin was assessed by comparing the proportion of intact cells after toxic challenge to the negative control without toxins (i.e., 100% of intact cells) using Student’s *t*-test. The effect of PVL between leucocyte sub-types was compared using Student’s *t*-test with Bonferroni adjustment. *, *p* < 0.05; **, *p* < 0.01; ***, *p* < 0.001; N.S., non-significant. Gr imm 2, granulocytic immature 2 cells; PMN, polymorphonuclear neutrophil cells; CD11b+ mo, CD11b+ monocytes; NK, natural killer; T NK, T natural killer.

The proportion of intact cells was significantly lower in the presence of PVL, compared to negative control without toxins, for PMN (34.3%, (_95%_CI): (25.2; 43.4), *p* < 0.001), Gr2 cells (75.2%, (_95%_CI): (59.9; 90.3), *p* < 0.01), CD11b+ monocytes (61.3%, (_95%_CI): (29.4; 93.2), *p* < 0.05), and NK (92.4%, (_95%_CI): (86.4; 98.4), *p* < 0.05; Figure 4). PVL-related cytotoxicity was significantly higher on PMN than on Gr2 cells (*t*-test, *p* < 0.001), but not significantly different between PMN and on CD11b+ monocytes (Figure 4 and Figure S2).

For the granulocytic myeloid-derived cells, there was a significant negative correlation between the proportion of intact cells and the C5aR expression level (Pearson test, r = −0.72, (_95%_CI): (−0.88; −0.43), *p* < 0.001; Figure S1b) and LukS-FITC binding (Pearson test, r = −0.76, (_95%_CI): (−0.89; −0.50), *p* < 0.001; Figure S1c). For CD11b+ monocytes, there was no significant correlation between the proportion of intact cells left and the C5aR expression level (Pearson test, *p* = 0.79; Figure S1b) or LukS-FITC binding (Pearson test, *p* = 0.32; Figure S1c).

#### 2.3.2. Effect of HlgABC

The proportion of intact CD11b+ monocytes (51.01%, (_95%_CI): (24.80; 77.21), *p* < 0.05) and intact NK (72.3%, (_95%_CI): (53.5; 91.1), *p* < 0.01) was significantly lower after of HlgABC challenge (Figure 4 and Figure S2). For NK cells, there was a significant negative correlation between the proportion of intact cells left after HlgABC challenge and the CXCR1 expression level (Pearson test, r = −0.66, (_95%_CI): (−0.91; −0.05), *p* < 0.05; Figure S3a). For CD11b+ monocytes, there was no negative correlation between the proportion of intact cells left after HlgABC challenge and the CXCR1 expression level (Pearson test, *p* = 0.13; Figure S3a), CCR2 expression level (Pearson test, r = 0.94, (_95%_CI): (0.76; 0.99), *p* < 0.001; Figure S3b), CXCR2 expression level (Pearson test, *p* = 0.68; Figure S3c), or C5aR expression level (Pearson test, *p* = 0.38; Figure S3d). For the other leucocyte populations, HlgABC had no significant effect, especially on PMN and granulocytic immature 2 (Figure 4).

## 3. Discussion

We report here that several bone marrow leucocyte populations expressed the target receptors of the staphylococcal leucocidin S sub-unit: C5aR for LuKS-PVL and HlgC [3,4], and CXCR1, CXCR2 and CCR2 for HlgA [4]. Interestingly, we observed that, within the granulocytic myeloid-derived leucocytes, the C5aR receptor was expressed according to cell maturity. Thus, we confirmed that C5aR is a marker of myeloid line maturation as previously described [10].

As expected, the leucocyte populations with a high level of C5aR expression (PMN, Gr2 cells and CD11b+ monocytes) also exhibited a high level of FITC LukS-PVL binding, since C5aR has been identified as being a LukS-PVL receptor [3]. Moreover, leucocyte populations that had the lowest C5aR expression (CD19+ B lymphocytes and the immature cells) did not bind FITC LukS-PVL. Incidentally, we observed that CD19+ B lymphocytes and the immature cells were not lysed by PVL, while leucocytes with a high density of C5aR (PMN, Gr 2 cells, and CD11b+ monocytes) were lysed by PVL at 0.5 µg/mL. This PVL concentration is known to result in lysis of circulating human PMN and circulating monocytes [11,12], and we can conclude that, to be lysed by PVL via C5aR, cells have to express an sufficient number of C5aR receptors at their surface. Nevertheless, we observed different behaviour between the granulocytic myeloid-derived leucocytes and monocytes; for the former, the PVL effect was correlated with C5aR expression level and with LukS-FITC binding, but not for monocytes for which an important variation between bone marrow donors was observed. This may be explained by the level of expression CD45, the PVL co-receptor [5], not precisely measured in this present study. Supplementary investigations should be performed to test these hypotheses, especially the measurement of the CD45 expression level by RMFI. Unexpectedly, we observed PVL activity on NK cells, while they did not express C5aR. This effect was very weak, probably without clinical impact. Nevertheless, this could suggest an indirect effect of PVL. Further investigations are warranted in order to explore this.

We have shown that staphylococcal Hlgs (HlgAB and HlgCB in the same assay) was able to lyse bone marrow leucocytes, in particular CD11b+ monocytes, which expressed the four tested receptors (C5aR, CXCR1, CXCR2, and CCR2), but not PMN or Gr2, which expressed only CXCR1 and C5aR. The observed effect of Hlg ABC on CD11b+ monocytes was very heterogeneous and was not correlated with the expression level of receptors for HlgA (CXCR1, CXCR2, and CCR2) or HlgC (C5aR). Spaan et al. observed that HlgAB and HlgCB resulted in circulating PMN and monocyte permeability [4]; the authors separately tested HlgAB and HlgCB, at a concentration of 63 nM (2 µg/L), which is 20 times more concentrated than herein (0.1 µg/L for each sub-unit), while sub-units S (HlgA and HlgC), and sub-unit F (HlgB) were incubated together with bone marrow cells. The difference in the toxin concentration tested could explain, at least in part, the discrepancies observed between the present study and that reported by Spaan et al. [4]. Another point to consider is the exposure to all subunits together. For instance, the affinity of HlgA binding for CXCR1, CXCR2 and CCR2 differs (respectively 5.69, 27.20, and 3.51 nM), and HlgC binds C5aR with an affinity constant of 5.64 nM [4]; consequently, we believe that, in the present study, CD11b+ monocytes were lysed by HlgAB pore-forming toxins through targeting of CXCR2, a receptor only expressed by these cells and for which HlgA has the highest affinity, HlgB being totally consumed to form pores with HlgA CD11b+ monocytes, which precluded HlgCB from forming pores on PMN or Gr2. Thus, this could explain why we have not observed HlgABC activity on other bone marrow cells expressing CXCR1, CCR2 and C5aR, especially PMN. Unexpectedly, and in conflict with the hypothesis mentioned above, we observed a variable, but significant, effect of HlgABC on NK cells, which express only CXCR1 and not CXCR2. This is the first time that the effect of staphylococcal Hlg on NK cells has been reported. Additional studies would be necessary to further explore this, particularly to understand why there is an important host susceptibility and what consequences this immune-modulatory effect could have in staphylococcal pathogenesis. Furthermore, the presence of a co-receptor for pore-forming by Hlg could also explain the lack of an HlgABC effect on PMN and Gr2 reported herein, and could be an interesting aspect to investigate further.

In conclusion, the results reported herein indicate that staphylococcal leucocidins, notably PVL, result in human bone marrow leucocyte lysis, notably of the most mature myeloid-derived leucocytes. This favours a peripheral origin of leukopenia occurring during necrotising pneumonia, because PVL affects neither stem cells nor the most immature myeloid-derived cells. However, the central destruction of mature myeloid-derived cells could worsen leukopenia during necrotising pneumonia; furthermore, a central component of leukopenia, through the HlgABC lytic effect on NK cells, is to be ruled out, as these cells play a key role in both innate and adaptive immunity and a recent study highlighted that a decrease in NK cells may lead to haematopoiesis failure in severe aplastic anaemia, which was characterised by severe pancytopenia and bone marrow failure [13]. The data presented suggest that a potentially interesting therapeutic strategy to improve the outcome of necrotising pneumonia, and notably to control leukopenia, could be to target and inhibit leucocidin receptors, in particular C5aR. PMX-53 is an antagonist of C5aR [14] that was initially developed to treat rheumatoid arthritis, for which it was not found to be effective [15]. Nevertheless, this study did report that PMX-53 treatment was well tolerated and that it inhibited human neutrophil activation by Ca5 in vitro [15]. Additionally, Choudhry et al. reported, in a mouse model of chronic *Escherichia coli* kidney infection, that blocking the C5a/C5aR axis, for example, by PMX-53, could be a way to reduce chronic kidney infection, at a time in which the multidrug resistance of Gram-negative bacteria is increasing [16]. Moreover, Tseng et al. noted that PMX-53 inhibited PVL binding to human PMN and thus PVL-induced cytotoxicity [17]. Although they also showed that PMX-53 did not enhance the outcomes of skin infection in “humanised” mice with human C5a receptors because of the alteration of innate immunity, this approach could hypothetically be favourable in the early management of life-threatening necrotising pneumonia management, when leukopenia is a major factor of a worse prognosis: the benefit of blocking leukocyte cytolysis could be transiently greater than the risk of impairing innate immunity.

## 4. Materials and Methods

### 4.1. Bone Marrow Selection

This study was conducted in accordance with the guidelines of the ethics committee of the participating hospital, Centre Hospitalier Lyon Sud (Lyon, France). All patients were informed of the use of their bone marrow for research purposes and were given the opportunity to refuse this according to French legislation. The leftovers of 11 bone marrow donors were selected to have normal bone marrow; 5 came from bone marrow donation, and 6 from a myelogram of patients affected by haematological disease, not affecting bone marrow leucocytes: 2 thrombocytopenic purpura cases, 2 cases of leukaemia post-treatment, and 2 regenerative anaemia and thrombopenia cases (sampling was performed as part of ongoing clinical trials NCT01773187, NCT02627261 and NCT03212053).

### 4.2. S. aureus Leucocidins

The staphylococcal leucocidins used in this study were recombinant proteins produced in *Escherichia coli* as previously described [18]: PVL, HlgsABC, and FITC-labelled LukS-PVL.

### 4.3. Expression of Bone Marrow Leucocytes Receptors

Analysis of bone marrow leucocyte surface receptor expression was performed by multi-parameter flow cytometry (MFC) using a Coulter Navios flow cytometer (Beckman Coulter, Brea, CA, USA). Briefly, the leucocyte populations were identified by multi-parameter combination using anti-CD56 phycoerythrin (IOtest clone N901; Beckman Coulter), anti-CD13 R Phycoerythrin-Texas Red^®^-X (IOtest clone SJ1D1; Beckman Coulter), anti-CD117 peridinin chlorophyll complex (PerCP)cy5.5 (IOtest clone 104D2D1; Beckman Coulter), anti-CD34 PerCPcy7 (IOtest clone 581; Beckman Coulter), anti-CD11b allophycocyanin (APC; IOtest clone Bear1; Beckman Coulter), anti-CD19 Alexa Fluor (AA) 700 (IOtest clone J3-119; Beckman Coulter), anti-CD3 AA750 (IOtest clone UCHT1; Beckman Coulter), anti-CD16 Pacific Blue (IOtest clone 3G8; Beckman Coulter), anti-CD45 Krome Orange (IOtest clone J.33; Beckman Coulter), and anti-CD117 APC (clone 104D2; BD Bioscience, Franklin Lakes, NJ, USA). After thawing, cells were incubated with the appropriate combination of monoclonal antibodies (MoAbs; 1 × 10^6^ total cells par tube), washed, and then analysed by flow cytometry (500,000 cells counted).

**Figure 5:**
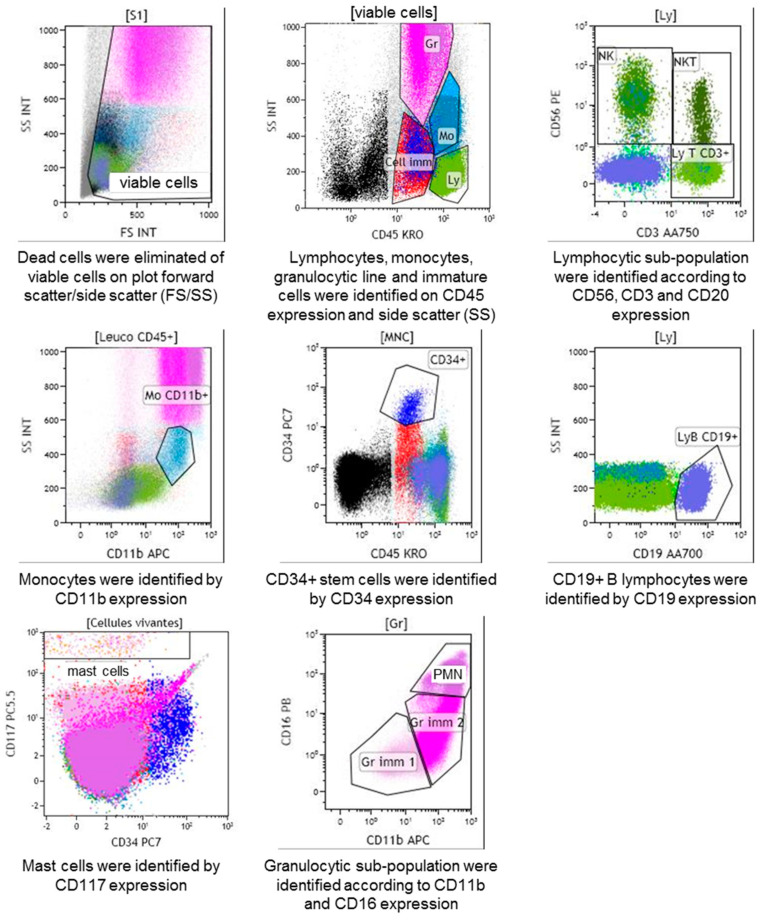
Analysis of bone marrow scatter. Gating strategy based on CD45/side scatter (SS) total cells gated from total forward scatter (FS)/SS viable cells, allowing the differentiation of the different bone marrow leucocyte subpopulation according to CD3, CD56, CD11, CD34, CD19, CD117, and CD16 expression. Gr, granulocytic myeloid-derived cells; Cell Imm, immature cells; CD34+, CD34+ stem cells; Gr imm1, granulocytic immature 1 cells; Gr imm 2, granulocytic immature 2 cells; PMN, polymorphonuclear neutrophil cells; Mo, monocytes; LyB CD19+, CD19 B lymphocytes; Ly T CD3+, T lymphocytes; NK, natural killer; T NK, T natural killer.

The gating strategy was based on CD45/side scatter (SSC) total cells gated from total forward scatter (FSC)/SSC viable cells, allowing the differentiation of immature cells, CD34+ stem cells, granulocytic myeloid-derived cells, monocytes, lymphocytes, and mast cells. Among the granulocytic myeloid-derived cells, three populations were distinguished based on the differential expression of CD11b and CD16: Gr1 cells, Gr2 cells, and PMN. Monocytes were characterised by CD11b expression and B lymphocytes by CD19 expression. Among the lymphocyte population, three populations were differentiated based on the expression of CD3 and CD56: T lymphocytes, which expressed CD3 but not CD56, NK, which expressed CD56 but not CD3, and T NK, which expressed both CD3 and CD56. Mast cells were characterised by CD117 expression (Figure 5).

For each patient, CXCR1, CXCR2, CCR2, and C5aR expression on leucocyte populations were analysed using MoAbs: anti-CXCR1 FITC (clone 42705; R&Systems, Minneapolis, MN, US), anti-CXCR2 FITC (clone 48311; R&Systems), anti-CCR2 FITC (clone 48607; R&Systems), anti-C5aR FITC (clone P12/1; Bio-Rad AbD Serotec, Hercules, CA, USA). Receptor expression was assessed by the RMFI, being the ratio between the median of the intensity of the fluorescence of specific monoclonal antibodies and the median of the intensity of the fluorescence of the isotypic control (Figure 1). RMFI ≥ 2 indicated that the population expressed the receptor. Both RMFI and fluorescence intensity were high, indicating a high expression of the target receptor.

### 4.4. Staphylococcal Leucocidins Effect on Leucocyte Populations

For each patient, LukS-PVL FITC binding assays, PVL (LukS-PVL and LukF-PVL) and Hlgs ABC cytotoxic assays were conducted separately. The bone marrow was incubated with 0.5 µg/mL of LukS-PVL FITC, 0.5 µg/mL of PVL (0.5 µg/mL lukS-PVL + 0.5 µg/mL lukF-PVL), or 0.1 µg/mL of Hlgs (0.1 µg/mL HlgsA + 0.1 µg/mL HlgsB + 0.1 µg/mL HlgsC) at room temperature for 20 min before being analysed by MFC. The leucocidin lytic effect was expressed as the ratio (presented as a proportion) between the proportion of each leucocyte sub-population in the gate challenged with toxins and the proportion of the same unchallenged sub-population (control; Figure 3). For each condition (control, PVL and Hlgs), 500,000 cells were counted with a flow cytometer.

### 4.5. Statistical Analysis

To compare the receptor expression and staphylococcal leucocidin effect between cell populations, Student’s *t*-test with Bonferroni adjustment was used; the effect on each cell population was compared to the negative control (100% intact) using Student’s *t*-test. Correlation was tested using the Pearson correlation test and tests were considered statistically significant at a *p* value < 0.05. Statistical analyses were performed using RStudio, version 0.99.893 (RStudio Team (2009–2016)). RStudio: Integrated Development for R. RStudio, Inc., Boston, MA, USA).

## Supplementary Materials

The following are available online at https://www.mdpi.com/2072-6651/12/11/725/s1, Figure S1: Correlation studies for the granulocytic myeloid-derived cells and CD11b+ monocytes. Figure S2: Effect of staphylococcal leucocidins PVL and HlgABC on bone marrow leucocytes. Figure S3: Correlation studies for CD11b+ monocytes and NK cells.

## References

[1] Lowy F.D. (1998). Staphylococcus aureus infections. N. Engl. J. Med..

[2] Grumann D., Nübel U., Bröker B.M. (2013). Staphylococcus aureus toxins—Their functions and genetics. Infect. Genet. Evol. J. Mol. Epidemiol. Evol. Genet. Infect. Dis..

[3] Spaan A.N., Henry T., van Rooijen W.J.M., Perret M., Badiou C., Aerts P.C., Kemmink J., de Haas C.J.C., van Kessel K.P.M., Vandenesch F. (2013). The staphylococcal toxin Panton-Valentine Leukocidin targets human C5a receptors. Cell Host Microbe.

[4] Spaan A.N., Vrieling M., Wallet P., Badiou C., Reyes-Robles T., Ohneck E.A., Benito Y., de Haas C.J.C., Day C.J., Jennings M.P. (2014). The staphylococcal toxins γ-haemolysin AB and CB differentially target phagocytes by employing specific chemokine receptors. Nat. Commun..

[5] Tromp A.T., Van Gent M., Abrial P., Martin A., Jansen J.P., De Haas C.J.C., Van Kessel K.P.M., Bardoel B.W., Kruse E., Bourdonnay E. (2018). Human CD45 is an F-component-specific receptor for the staphylococcal toxin Panton-Valentine leukocidin. Nat. Microbiol..

[6] Gillet Y., Issartel B., Vanhems P., Fournet J.-C., Lina G., Bes M., Vandenesch F., Piémont Y., Brousse N., Floret D. (2002). Association between Staphylococcus aureus strains carrying gene for Panton-Valentine leukocidin and highly lethal necrotising pneumonia in young immunocompetent patients. Lancet.

[7] Gillet Y., Vanhems P., Lina G., Bes M., Vandenesch F., Floret D., Etienne J. (2007). Factors predicting mortality in necrotizing community-acquired pneumonia caused by Staphylococcus aureus containing Panton-Valentine leukocidin. Clin. Infect. Dis. Off. Publ. Infect. Dis. Soc. Am..

[8] Diep B.A., Chan L., Tattevin P., Kajikawa O., Martin T.R., Basuino L., Mai T.T., Marbach H., Braughton K.R., Whitney A.R. (2010). Polymorphonuclear leukocytes mediate Staphylococcus aureus Panton-Valentine leukocidin-induced lung inflammation and injury. Proc. Natl. Acad. Sci. USA.

[9] Szmigielski S., Jeljaszewicz J., Wilczynski J., Korbecki M. (1966). Reaction of rabbit leucocytes to staphylococcal (Panton-Valentine) leucocidin in vivo. J. Pathol. Bacteriol..

[10] Werfel T., Oppermann M., Schulze M., Krieger G., Weber M., Götze O. (1992). Binding of fluorescein-labeled anaphylatoxin C5a to human peripheral blood, spleen, and bone marrow leukocytes. Blood.

[11] Löffler B., Hussain M., Grundmeier M., Brück M., Holzinger D., Varga G., Roth J., Kahl B.C., Proctor R.A., Peters G. (2010). Staphylococcus aureus panton-valentine leukocidin is a very potent cytotoxic factor for human neutrophils. PLoS Pathog..

[12] Holzinger D., Gieldon L., Mysore V., Nippe N., Taxman D.J., Duncan J.A., Broglie P.M., Marketon K., Austermann J., Vogl T. (2012). Staphylococcus aureus Panton-Valentine leukocidin induces an inflammatory response in human phagocytes via the NLRP3 inflammasome. J. Leukoc. Biol..

[13] Liu C., Li Z., Sheng W., Fu R., Li L., Zhang T., Wu Y., Xing L., Song J., Wang H. (2014). Abnormalities of quantities and functions of natural killer cells in severe aplastic anemia. Immunol. Investig..

[14] Haynes D.R., Harkin D.G., Bignold L.P., Hutchens M.J., Taylor S.M., Fairlie D.P. (2000). Inhibition of C5a-induced neutrophil chemotaxis and macrophage cytokine production in vitro by a new C5a receptor antagonist. Biochem. Pharmacol..

[15] Vergunst C.E., Gerlag D.M., Dinant H., Schulz L., Vinkenoog M., Smeets T.J.M., Sanders M.E., Reedquist K.A., Tak P.P. (2007). Blocking the receptor for C5a in patients with rheumatoid arthritis does not reduce synovial inflammation. Rheumatol. Oxf. Engl..

[16] Choudhry N., Li K., Zhang T., Wu K.-Y., Song Y., Farrar C.A., Wang N., Liu C.-F., Peng Q., Wu W. (2016). The complement factor 5a receptor 1 has a pathogenic role in chronic inflammation and renal fibrosis in a murine model of chronic pyelonephritis. Kidney Int..

[17] Tseng C.W., Biancotti J.C., Berg B.L., Gate D., Kolar S.L., Müller S., Rodriguez M.D., Rezai-Zadeh K., Fan X., Beenhouwer D.O. (2015). Increased Susceptibility of Humanized NSG Mice to Panton-Valentine Leukocidin and Staphylococcus aureus Skin Infection. PLoS Pathog..

[18] Perret M., Badiou C., Lina G., Burbaud S., Benito Y., Bes M., Cottin V., Couzon F., Juruj C., Dauwalder O. (2012). Cross-talk between Staphylococcus aureus leukocidins-intoxicated macrophages and lung epithelial cells triggers chemokine secretion in an inflammasome-dependent manner. Cell. Microbiol..

