# Supplementary material for: Staphylococcal Panton–Valentine Leucocidin and Gamma Haemolysin Target and Lyse Mature Bone Marrow Leucocytes
Source: Toxins (Basel). 2020 Nov 20;12(11):725. doi: 10.3390/toxins12110725 (PMC7699679; doi:10.3390/toxins12110725)
Supplement: Supplementary file 1 [file toxins-12-00725-s001.pdf]

Supplementary Materials: Staphylococcal Panton–Valentine Leucocidin and Gamma Haemolysin Target and Lyse Mature Bone Marrow Leucocytes

Elisabeth Hodille [1,2,*], Adriana Plesa [3], Eve Bourrelly [3], Lucie Belmont [3], Cédric Badiou [2], Gerard Lina [1,2,4] and Oana Dumitrescu [1,2,4]

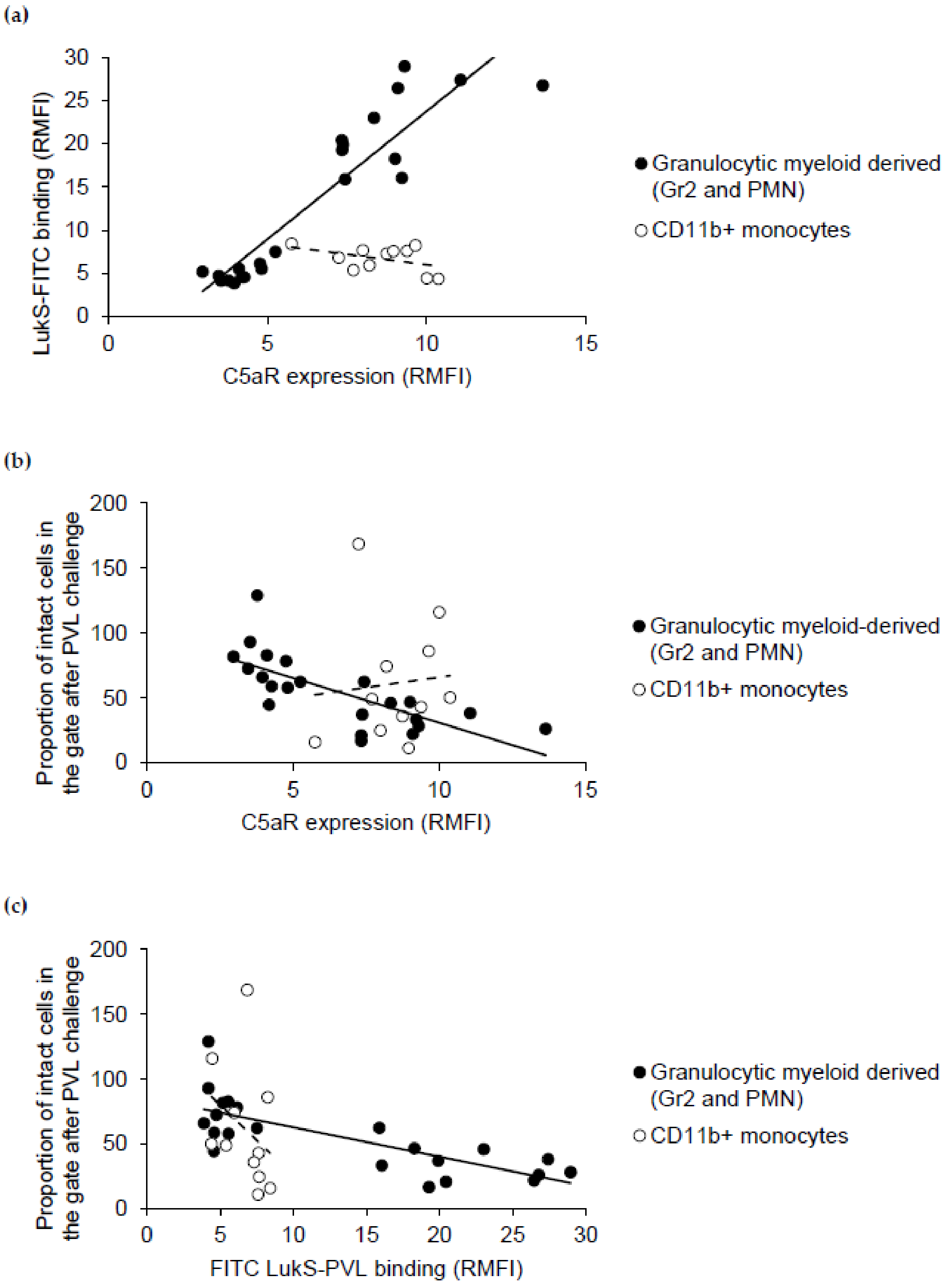

Figure S1. Correlation studies for the granulocytic myeloid-derived cells and CD11b+ monocytes. (**a**) Correlation between C5aR expression and LukS-FITC binding. (**b**) Correlation between C5aR expression and PVL-related cytotoxicity. (**c**) Correlation between FITC LukS-PVL binding and PVL-related cytotoxicity.

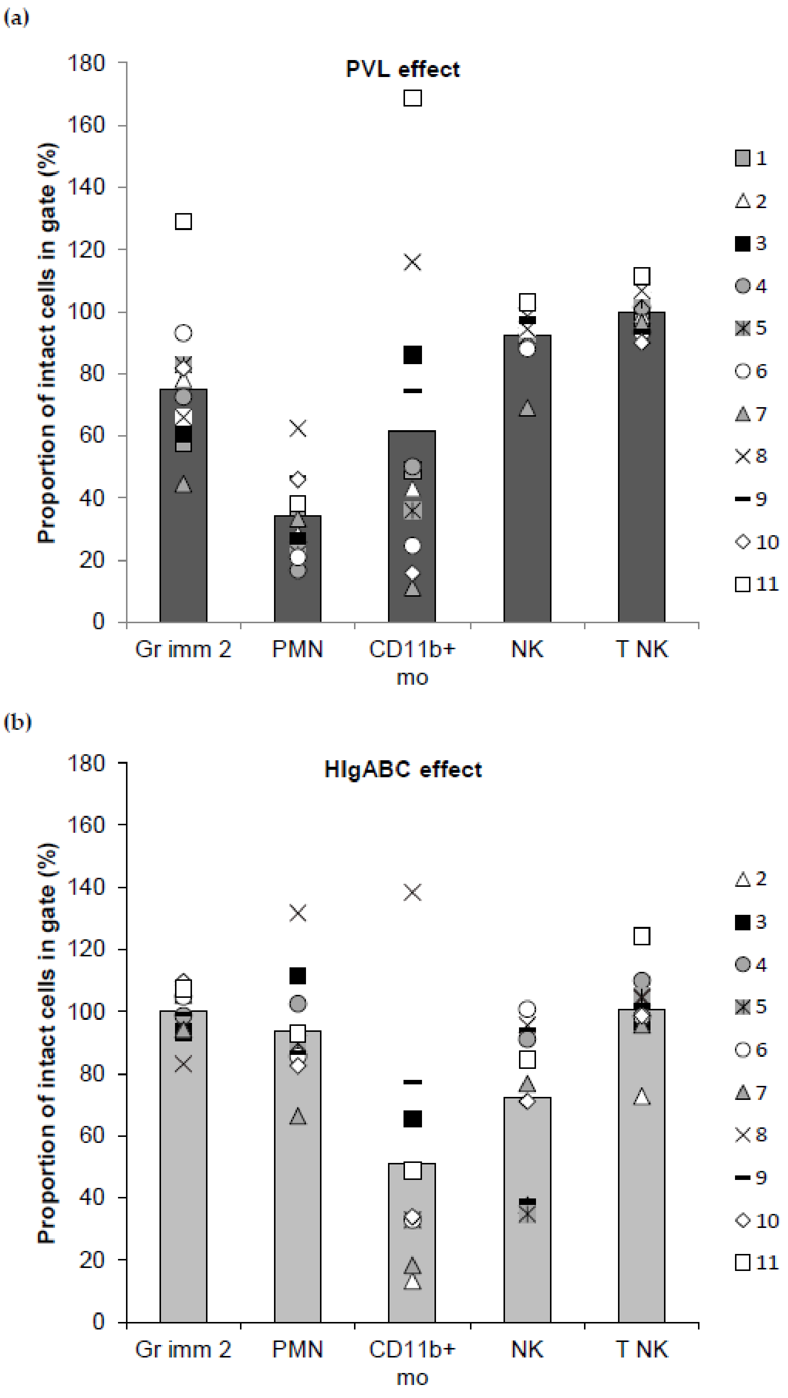

Figure S2. Effect of staphylococcal leucocidins PVL (**a**) and HlgABC (**b**) on bone marrow leucocytes, represented by the proportion of intact cells after incubation with toxins on the corresponding gate. Bar plot corresponding to mean of 11 bone marrows for PVL (**a**) and 10 bone marrows for HlgABC (**b**). Each symbol corresponding to 1 bone marrow. Gr imm 2, granulocytic immature 2 cells; PMN, polymorphonuclear neutrophil cells; CD11b+ mo, CD11b+ monocytes; NK, natural killer; T NK, T natural killer.

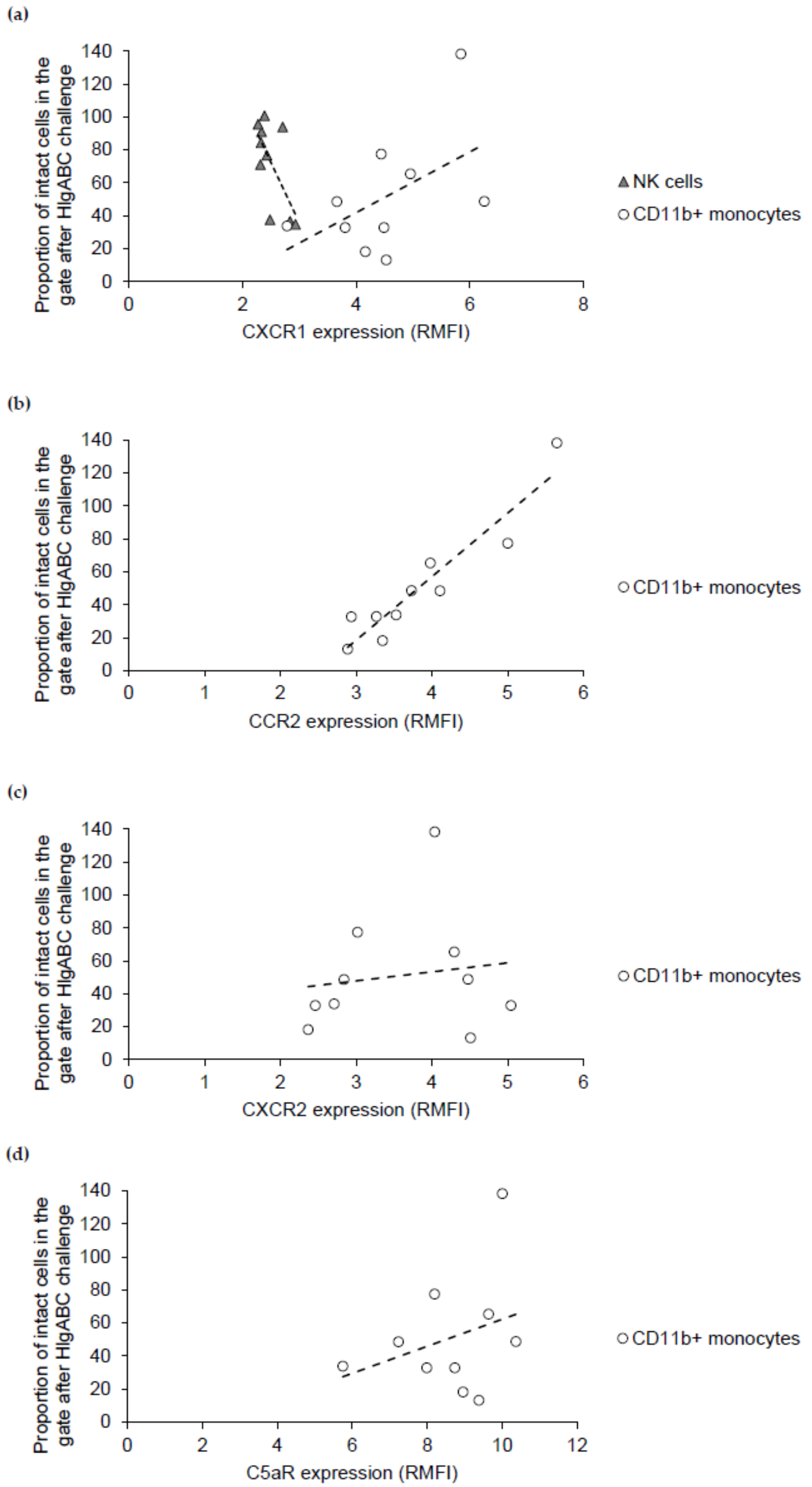

Figure S3. Correlation studies for CD11b+ monocytes and NK cells. (**a**) Correlation between CXCR1 expression and HlgABC-related cytotoxicity. (**b**) Correlation between CCR2 expression and HlgABC-related cytotoxicity. (**c**) Correlation between CXCR2 expression and HlgABC-related cytotoxicity. (**d**) Correlation between C5aR expression and HlgABC-related cytotoxicity.
